# Using blood biomarkers and ophthalmological indicators of optical coherence tomography and angiography for the diagnosis of fundus lesions in patients with diabetes mellitus

**DOI:** 10.3389/fcdhc.2025.1499344

**Published:** 2025-04-08

**Authors:** Fanny Huang, Miaomiao Yu, Laura Huang, Ruikang K. Wang, Theodore Leng, Sophia Y. Wang, Yaping Joyce Liao

**Affiliations:** ^1^ Department of Ophthalmology, Stanford University School of Medicine, Stanford, CA, United States; ^2^ Department of Neurology, Stanford University School of Medicine, Stanford, CA, United States; ^3^ Department of Ophthalmology, University of Washington, Seattle, WA, United States

**Keywords:** diabetic retinopathy, ophthalmic imaging, optic nerve, ophthalmic coherence tomography, vision loss

## Abstract

**Purpose:**

To assess long-term ophthalmic and clinical blood test changes in patients with different severities of diabetic retinopathy (DR).

**Methods:**

We performed a longitudinal case-control study of 130 patients with diabetes mellitus (DM) and 67 controls, including visual acuities from 2,201 eye clinic visits and 44,833 blood tests. We also analyzed optic disc and macular structure and vasculature using optical coherence tomography (OCT) and angiography (OCTA).

**Results:**

Ninety-one percent of eyes in diabetic patients had stable visual acuity (better than 20/40) over 7 years. Cluster analysis revealed most prominent blood test changes in the DM included elevated glucose and hemoglobin A1c and evidence of nephropathy. Optic disc OCTA was most correlated with OCT in the superior and inferior quadrants. Notably, peripapillary and macular OCTA measurements revealed evidence of microvascular drop out even in those with DR grade 0.

**Conclusions:**

Majority of patients with DM monitored by physicians maintained good visual acuity over years. Ophthalmic imaging revealed evidence of early vascular changes even in patients without evidence of DR on clinical exam and color fundus imaging. In addition to ophthalmic functional and structural assessments, clinical blood tests for renal function are also important early biomarkers of end organ damage in DM.

## Introduction

Diabetic retinopathy (DR) is the most common cause of vision loss in the working population and one of the most feared consequences of diabetes mellitus (DM) (1). DR is characterized by a combination of metabolic syndrome-induced retinal and optic nerve vascular disease and microaneurysms, leading to ischemia, hemorrhage, edema, and neurodegeneration (1–3). Given that the retina is one of the most metabolically active tissues in the body, with its blood supply from the superficial capillary plexus of the superficial retinal layer, it’s no surprise that DR is highly correlated with end-organ damage, peripheral neuropathy, and cognitive decline (1, 3–5). DR is graded over 5 stages, from no vasculopathy (DR0) to non-proliferative DR (DR1-3), and proliferative DR (DR4) (1, 2, 5). Proliferative DR is defined by neovascularization and chronic hyperglycemia, leading to retinal neurodegeneration through inflammation and increased oxidative stress. Important unmet needs in the field include the lack of good early diagnostic and prognostic biomarkers of DR prior to the development of vision loss (5, 6). Thus, noninvasive ophthalmic measurements may be the most accessible early biomarker for diabetic end-organ damage (7).

Optical coherence tomography (OCT) imaging is routinely used to assess retinal thickness and pathology in patients with DR in the clinical setting. However, there is still a lack of imaging biomarker for DR. Common OCT findings in DR include macular edema, which increases retinal thickness, and neurodegeneration, which leads to retinal thinning. Macular edema and thinning can also occur simultaneously, resulting in no change in retinal thickness (8, 9). Optical coherence tomography angiography (OCTA) is a highly promising, novel, noninvasive imaging technique, but is limited by segmentation algorithms needed for quantitative assessment (10, 11). The most severely affected part of the retina in DR is the inner retina (8, 9), which is supplied by the superficial capillary plexus, which is easily measured and quantified *in vivo* using OCTA (8, 10). Such imaging is now routinely performed in 10 minutes without pupillary dilation in the eye clinic (7). Limitations of the OCTA study in DR include: 1) difficulty of obtaining high quality images and limitation of the analysis software, which can typically only measure vessel area density (VAD) and 2) lack of correlation between OCTA measurements and clinical assessments of DM itself. In this study, we used OCTA imaging on patients in order to better understand the ophthalmic vascular changes observed in patients with diabetes. The novelty of this study lies within the limited use of OCTA in current clinical settings for patients with diabetes to examine early retinal changes. The necessity of this study were to improve early clinical management for patients with diabetes by detecting early vascular changes on OCTA that correlate with progressive retinal disease due to underlying diabetes.

The aims of this study were to assess long-term ophthalmic and clinical blood test changes in patients with different severities of diabetic retinopathy through analyzing OCTA and clinical laboratory data from baseline and most recent follow up.

## Methods

In this study, we performed deep phenotyping of visual function and structure and in patients with DM and compared analysis of clinical blood tests with that of severity of DR. We analyzed over 300 DM and control eyes using >100 ophthalmic parameters per eye and assay longitudinal visual outcomes 4-7 years later. Our study will provide better understanding of the management of patients with DR beyond that of ophthalmic measurements.

### Study participants

This is a cross-sectional longitudinal observational study, approved by the Stanford Institutional Review Board and adherent to the Declaration of Helsinki, in compliance with the Health Insurance Portability and Accountability Act.

We created a large database of DM patients using the STAnford Research Repository (STARR) database (12), which contains electronic health records for patients in the Stanford healthcare system. All study participants were first seen at the Byers Eye Institute at Stanford University between 2014 and 2018, and OCTA was separately acquired for these patients. The number of patients was not calculated prior to this study.

Inclusion criteria included any patient diagnosed with DM (including Type 1 and Type 2) with a normal anterior segment and clear media, which was determined by a single retina specialist. Exclusion criteria included presence of a nuclear, cortical, or posterior subcapsular cataract that was grade 3 or higher, epiretinal membrane, macular hole, vitreomacular traction, glaucoma, staphyloma, or tractional retinal detachment as determined by a single retina specialist (TL). 130 patients with DM (218 eyes) were included in this study. All control subjects (67 healthy controls, 95 eyes) were volunteers without evidence of retinal vascular or optic nerve disease as determined by a single neuro-ophthalmologist (JL).

All diabetic patients were graded according to the International Clinical Diabetic Retinopathy Disease Severity Scale at baseline, throughout their ophthalmic care, and follow-up, that included no retinopathy (DR0), non-proliferative diabetic retinopathy (NPDR) categorized into mild (DR1), moderate (DR2), and severe (DR3); and proliferative diabetic retinopathy (PDR) (DR4) (1). Diabetic macular edema was graded as no center-involving diabetic macular edema (Grade 0), presence of active center-involving diabetic macular edema (Grade 1), and quiescent center-involving diabetic macular edema (Grade 2).

We extracted a comprehensive list of patient demographics using diagnosis test codes (**Table 1**). Custom scripts using ICD9/ICD10 codes were used to extract patient demographics, past medical history (hypertension, hypercholesterolemia, macular degeneration, glaucoma, and Type 2 DM), and all relevant clinical and laboratory tests (HbA1c, serum glucose, albumin, creatinine, estimated glomerular filtration rate, blood urea nitrogen, protein, globulin, alkaline phosphate, alanine transaminase, aspartate transferase, bilirubin, potassium, calcium, chloride, sodium, carbon dioxide, hemoglobin, red blood cell distribution width, hematocrit, high density lipoprotein cholesterol, low density lipoprotein cholesterol, non-high density lipoprotein cholesterol, and total cholesterol) between 2014 and 2022 (85% with multiple follow-up measurements over 4-7 years) using Stanford’s electronic health records database. These clinical biomarkers were extracted within the timeframe of OCTA measurements, as OCTA measurements were from 2018. LogMAR is calculated as -Log(Snellen numerator/Snellen denominator), where LogMAR (20/20) = 0.0 and LogMAR (20/200) = 1 (13).

**Table 1:** Demographic information of control (Ctrl) groups and diabetic mellitus (DM).

	Ctrl (N=15)	DM (N=83)	*p*-value
Mean Age in Years (SD)	57.5 (13.0)	60.5 (12.9)	Welch’s t (19.33) = -0.83, *p =* .416
Female	8 (53.3%)	42 (50.6%)	X^2^ (1) = 0.04, *p =* .846
Male	7 (46.7%)	41 (49.4%)	
White	10 (66.7%)	30 (36.1%)	X^2^ (6) = 11.76 *p =* .068
Asian	2 (13.3%)	24 (28.9%)	
Black or African American	0 (0.00%)	5 (6.0%)	
Native Hawaiian or Other Pacific Islander	0 (0.00%)	1 (1.2%)	
Other	2 (13.3%)	22 (26.5%)	
Unknown	1 (6.7%)	1 (1.2%)	
Non-Hispanic/Non-Latino	13 (86.7%)	68 (81.9%)	X^2^ (2) = 6.58 *p =* .037
Hispanic/Latino	1 (6.7%)	15 (18.1%)	
Unknown	1 (6.7%)	0 (0.0%)	

### OCT and OCTA acquisition, processing, and quantification

OCT and OCTA performed at the Byers Eye Institute, Stanford University was collected between December 1, 2017 and October 31, 2018. From the ophthalmic imaging database Forum, we extracted a wide-range of peripapillary and macular OCT imaging metrics. OCT and OCTA images were acquired using spectral-domain Cirrus HD-OCT (AngioPlex, 68 kHz, Model 5000; Carl Zeiss Meditec In., Germany). To measure OCT RNFL, we performed the Optic Disc Cube scan pattern acquiring 200 horizontal scan lines each composed of 200 A-scans. We quantified average thickness of RNFL using a circle with 3.46 mm diameter centered on the optic disc and quadrant RNFL thickness (superior, nasal, inferior and temporal) (**Table 2**). To measure macular ganglion cell complex, we used Zeiss automatic segmentation software to quantify mean macular ganglion cell complex and thickness in 6 sectors: superior, superior temporal, superior nasal, inferior temporal, inferior nasal, and inferior. Visual field testing was obtained using matching, which was performed at the same time as OCT/OCTA acquisition. Reliability of visual field testing was accounted by Zeiss Humphrey Field Analyzer 3.

**Table 2:** Mean and standard deviations of OCT measurements in RNFL quadrants and GCC sectors for each DR grade.

	Section	DR0	DR1	DR2	DR3	DR4
RNFL	T	64.52 (12.44)	59.61 (13.42)	59.79 (13.09)	67.18 (16.35)	62.03 (9.46)
	S	115.37 (18.50)	111.39 (17.06)	101.77 (25.72)	106.75 (22.40)	106.44 (21.60)
	N	77.50 (59.55)	68.83 (12.36)	63.59 (8.47)	76.17 (23.25)	61.35 (11.05)
	I	119.56 (18.65)	114.78 (11.97)	109.08 (18.13)	106.03 (27.96)	106.57 (25.19)
	Whole	92.19 (13.80)	88.61 (10.08)	84.37 (13.79)	93.87 (18.65)	84.52 (14.98)
GCC	ST	84.51 (10.57)	82.20 (11.50)	83.94 (10.21)	95.75 (5.12)	74.53 (23.07)
	S	81.36 (9.02)	77.20 (9.46)	74.62 (16.25)	85.38 (9.62)	68.20 (22.84)
	SN	85.69 (11.51)	82.05 (11.84)	75.71 (26.83)	90.38 (9.35)	73.53 (26.91)
	IT	82.29 (10.83)	79.95 (10.88)	82.12 (11.36)	97.00 (11.88)	71.47 (22.56)
	I	79.69 (10.08)	76.35 (9.80)	76.31 (17.24)	78.75 (10.26)	65.60 (25.25)
	IN	82.16 (11.70)	76.70 (10.55)	72.41 (24.40)	85.38 (8.11)	69.87 (22.12)
	Whole	80.74 (7.87)	77.95 (5.93)	73.71 (20.93)	87.50 (6.57)	70.87 (20.95)

To measure OCTA, we used 3×3 mm^2^ square scans of the optic disc were obtained using the FastTrac eye tracking system for OCTA imaging. Algorithms for optical microangiography (OMAG) based automatic segmentation of the raw OCTA data were used to get superficial retinal layer (SRL) *en face* peripapillary OCTA image, which includes vasculature from the ganglion cell layer and nerve fiber layer. Only images with signal strength index > 7 were used for analysis. We use validated customized quantification scripts using MATLAB software (coded with MATLAB R2016a; MathWorks, Natick, MA) to quantify OCTA images based on the modification of previous algorithm. For each OCTA image, we removed large vessels from the images, perform Hebbian and threshold transformation, and calculated 6 vessel parameters of the peripapillary retina, including vessel area density (VAD), vessel skeleton density (VSD), vessel perimeter index (VPI), vessel complexity index (VCI), vessel diameter index (VDI), and flux index. VAD reflects the proportion of the OCTA image that is occupied by blood vessels. VSD also reflects the OCTA image occupied by blood vessels, but adjusts for variability in different eyes by removing outer pixels of individual vessels so that each individual vessel is quantified by a single line of pixels. VDI is the fraction of VSD divided by VSD, indicating average vessel diameter in an OCTA image. VPI represents the quantification of pixels in vessel perimeters as a ratio to the total area of the OCTA image. VCI quantifies vessel tortuosity by dividing all of the pixels enclosed by vessel perimeters by all of the pixels in a vessel area. Flux represents perfusion of the vessel by measuring the number of red blood cells moving through a segment of a vessel per unit time. These parameters were used in this study as they are the most commonly used quantification parameters of OCTA imaging and also allows to quantify neovascularization in varying severities of DR.

For the optic disc OCTA measurements, we analyzed an annulus region of interest with outer diameter of 1.375 mm and inner diameter of 0.5 mm centered around the disc and the macula and calculate an average for the eye as well as average for 4 disc quadrants: superior, nasal, inferior, and temporal. For the macula, we used an annulus region of interest with outer diameter of 1.375 mm and inner diameter of 0.75 mm centered around the foveal avascular zone and calculated average macular vessel parameters and OCTA parameters in 6 sectors: superior, superior temporal, superior nasal, inferior temporal, inferior nasal, and inferior.

### Statistical analysis

The data were analyzed by custom Python version 3.9.6 (Python Software Foundation, 2021. Retrieved from https://www.python.org). and R scripts (R Foundation for Statistical Computing, Vienna, Austria). Quantitative continuous variables were calculated as mean ± standard error (SE) or median (95% confidence interval). A two-tailed Mann-Whitney U test was used to compare the means of two groups of continuous variables. The frequencies of categorical variables were compared using Chi-square test. Kruskal-Wallis H test was performed to determine if there are statistically significant differences among more than 2 groups of independent variables. Spearman correlation test was performed to determine the correlation between OCT, OCTA and visual field MD parameters. The correlation coefficients between 0 and 0.3 indicate weak correlation, between 0.3 and 0.7 indicate moderate correlation, and between 0.7 and 1 indicate high correlation. A value of *P <*0.05 was considered statistically significant.

## Results

### Study goal

**Figure 1:**
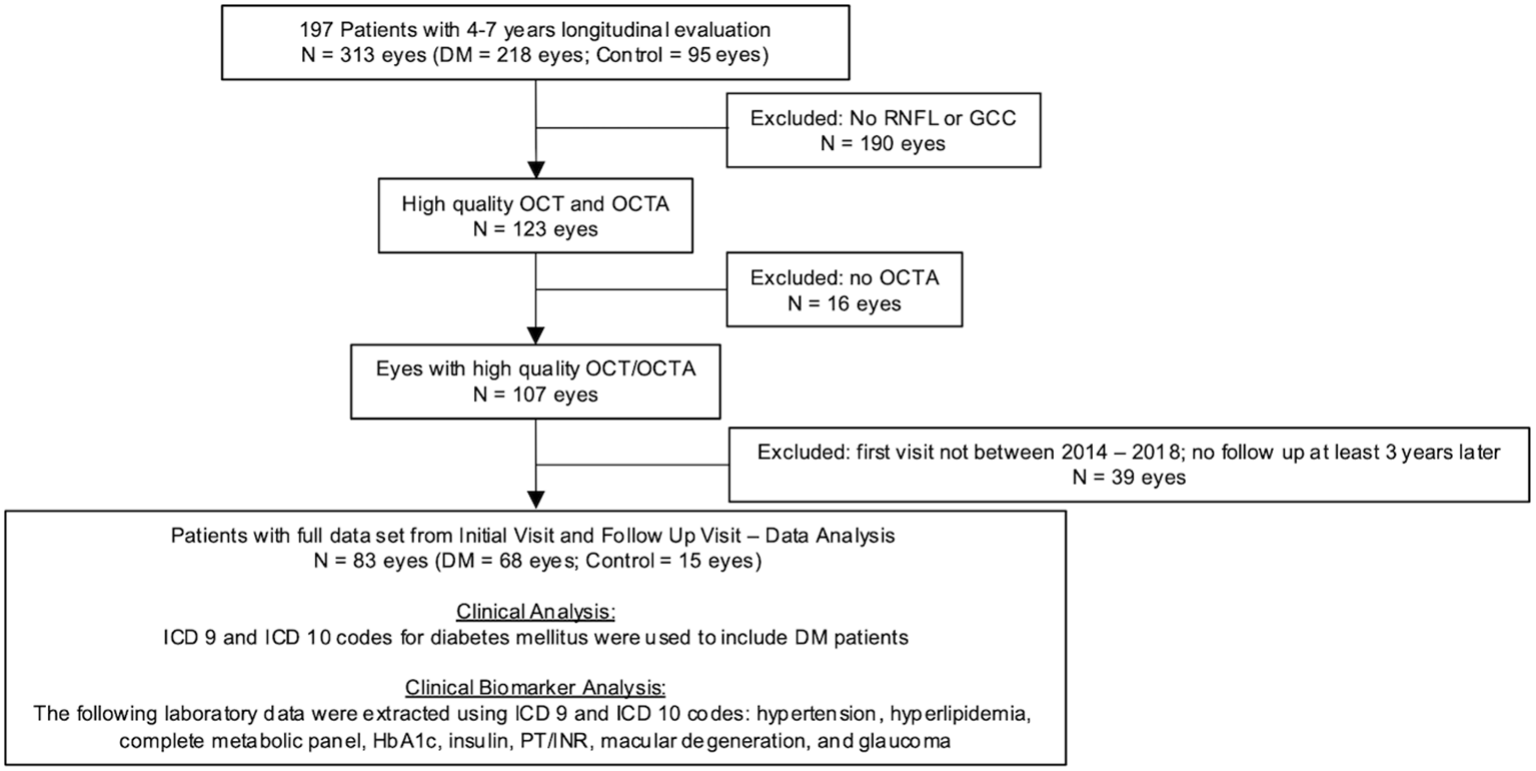
Flowchart of ophthalmic imaging analysis in patients with diabetes mellitus and control. Of the 218 Dm eyes, 181 had visual acuity data and 71 of those patients had follow-up visual acuity data 4-7 years after their initial visit.

Longitudinal evaluation of 44,833 data points of various blood laboratories and 2,201 eye clinic encounters that included visual acuity over 7 years revealed hyperglycemia, elevated HbA1c, and worsening renal function tests were consistent in patients with moderate to severe diabetic retinopathy. **Figure 1** outlines the stepwise approach of this study to collect and analyze data.

### DM patients with more severe retinopathy had worse visual acuity, although the majority of eyes had relatively stable visual acuity over 4-7 years under ophthalmic care

**Figure 2:**
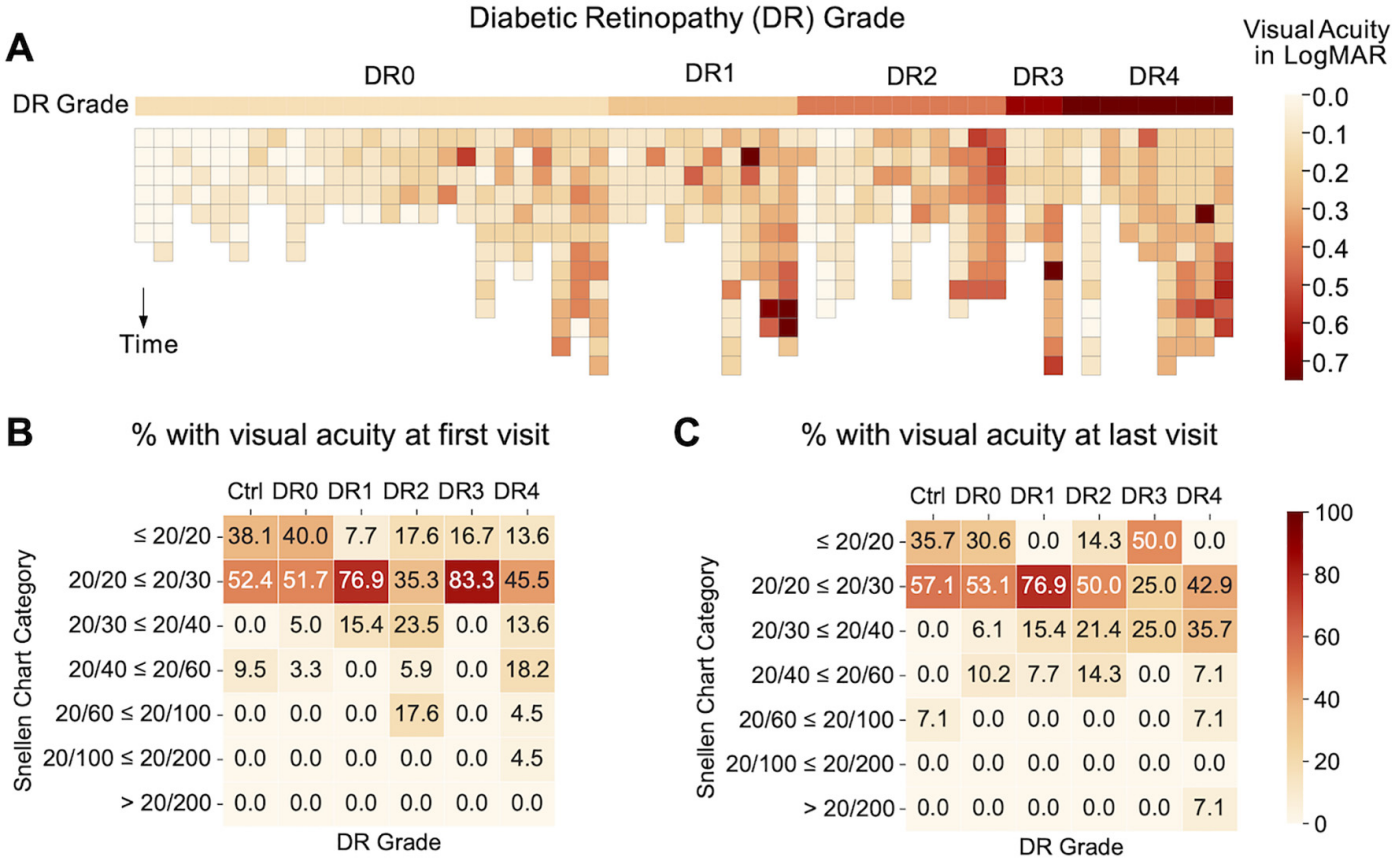
**(A)** Heatmap illustrating visual acuity of individuals within each diabetic retinopathy grade over time. Subjects are arranged by average LogMAR within each DR grade and darker colors are used to indicate a worsening of visual acuity. The pattern indicates an association between severity of DR grades and visual acuity: DR0 subjects have fewer cases of visual acuity worse than 20/60 as compared to other DR grades. **(B)** Percentage (%) of subjects with different categories of visual acuity by Snellen chart at first clinic visit (baseline) and **(C)** last clinic visit (follow-up at least 4-7 years after baseline).

At baseline, where patients’ DR grade were first documented at Byers Eye Institute, analysis of 2,201 eye clinic encounters that included visual acuity revealed that 90% of healthy controls had visual acuity of 20/20 – 20/30 at baseline and 93% of healthy controls had 20/20 – 20/30 visual acuity at most recent follow up visit. Longitudinal comparisons of visual acuity over 4-7 years revealed that of the DM eyes, 94 eyes had repeat measurements over 4-7 years. Analysis of best corrected visual acuity over 4-7 years revealed that the majority of patients had relatively stable visual acuity (**Figure 2**). DR visual acuity ranges from five eyes from DR3 and DR4 (8.62%) worsened over 4-7 years, but 53 eyes (91.38%) of DR eyes had relatively stable visual acuity over 4-7 years. Relatively stable visual acuity was defined as changing fewer than two lines on the Snellen Chart Category while worsened visual acuity was defined as changing two or more lines on the Snellen Chart Category.

### Blood tests revealed a correlation of severity of diabetic retinopathy

**Figure 3:**
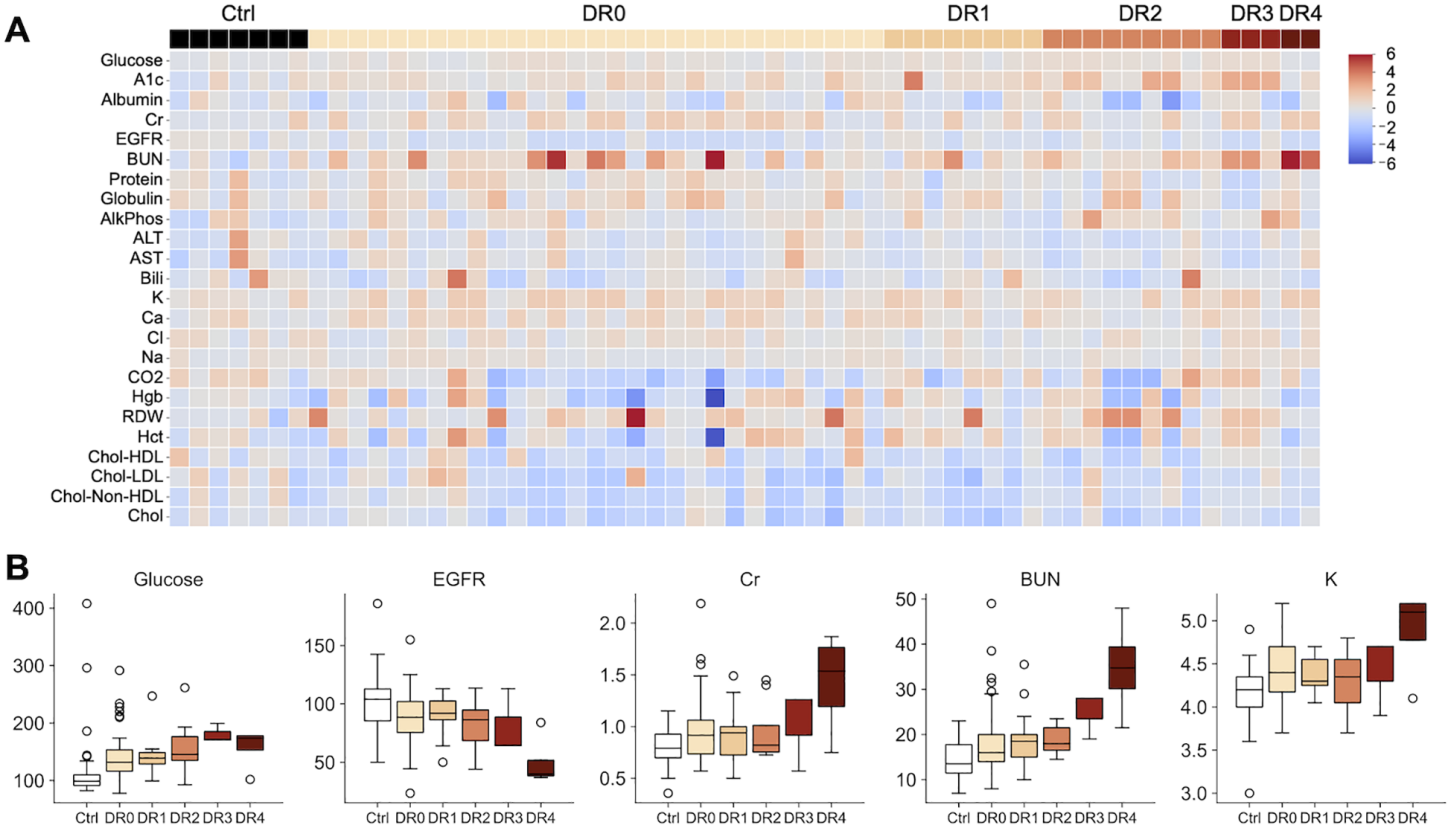
**(A)** Heatmap illustrating 45,221 data points of clinical blood tests of healthy controls (Ctrl) and individuals with different diabetic retinopathy grades (DR0-4). Higher A1c and renal function tests are more common in patients with moderate to severe DR. (DR 2: moderate DR. DR3: severe non-proliferative DR. DR 4: severe proliferative DR with neovascularization.) Values are standardized by the distribution of the control group. **(B)** Boxplots illustrating the unstandardized blood test values of glucose, EGFR, Cr, BUN and K. Glucose, Cr, BUN and K show an increasing trend with DM severity, while EGFR decreases. The axis for Cr is restricted at 2.5 for presentation purposes: one DR4 patient had a Cr reading of 7.0.

We next analyzed 44,833 data points of blood tests of 95 DM patients vs 10 controls over 4-7 years and showed that patients with moderate (DR grade 3) to severe (DR grade 4) diabetic retinopathy showed worsening renal insufficiency over time (**Figure 3**, **Table 3**) with increased DR severity. Glucose and HbA1c were globally significantly elevated in DR0-4 compared with controls (**Figure 3**, **Table 3**). Renal function including mean serum BUN, Cr, EGFR, and K worsened. DR3 and DR4 patients had greatest increase in mean BUN levels (DR3: 25.00 ± 5.20 mg/dL and DR4 39.60 ± 14.38 mg/dL; control: 14.66 ±4.29 mg/dL), Cr (DR4: 2.54 ±2.54 mg/dL; control: 0.81 ± 0.19 mg/dL), and EGFR (DR4: 41.80 ± 27.18 mL/min/1.73m^2^; control: 101.26 ± 25.30 mL/min/1.73m^2^), compared to that of healthy controls (**Table 3**).

**Table 3:** Mean and standard deviation of results from lab tests for the controls and patients of each diabetic retinopathy grade.

Blood Tests	Control	DR0	DR1	DR2	DR3	DR4
Glucose	118.60 (63.41)	140.80 (39.41)	143.08 (35.91)	156.14 (45.73)	180.50 (16.45)	147.30 (38.59)
A1c	6.04 (1.42)	6.91 (1.15)	7.60 (1.62)	7.60 (1.30)	9.53 (0.12)	7.65 (1.44)
Albumin	4.18 (0.33)	4.10 (0.34)	3.96 (0.41)	3.79 (0.53)	4.10 (0.35)	3.87 (0.27)
Cr	0.81 (0.19)	0.95 (0.30)	0.90 (0.29)	0.95 (0.26)	1.03 (0.40)	2.54 (2.54)
EGFR	101.26 (25.30)	87.56 (23.54)	89.62 (18.76)	81.36 (21.80)	80.67 (28.00)	41.80 (27.18)
BUN	14.66 (4.29)	18.30 (7.57)	19.00 (7.15)	18.77 (3.10)	25.00 (5.20)	39.60 (14.38)
Protein	7.32 (0.56)	7.45 (0.35)	7.32 (0.36)	7.34 (0.43)	6.77 (0.29)	7.24 (0.47)
Globulin	3.14 (0.65)	3.40 (0.58)	3.40 (0.65)	3.54 (0.78)	2.73 (0.58)	3.30 (0.58)
Alk Phos	74.26 (23.74)	76.51 (19.39)	77.58 (32.13)	86.64 (24.54)	98.00 (38.11)	80.40 (23.54)
ALT	31.96 (16.23)	30.79 (11.88)	31.08 (15.04)	22.00 (8.23)	25.17 (12.41)	32.60 (17.67)
AST	25.14 (8.47)	24.03 (6.01)	24.58 (7.32)	22.64 (4.79)	16.83 (3.18)	28.10 (12.28)
Bili	0.53 (0.22)	0.50 (0.28)	0.51 (0.27)	0.45 (0.32)	0.50 (0.00)	0.40 (0.12)
K	4.16 (0.35)	4.40 (0.36)	4.36 (0.21)	4.27 (0.38)	4.43 (0.46)	4.86 (0.46)
Ca	9.23 (0.34)	9.44 (0.38)	9.30 (0.35)	9.16 (0.43)	9.30 (0.17)	9.48 (0.48)
Cl	102.06 (2.73)	102.08 (2.49)	102.12 (2.00)	102.36 (2.51)	104.17 (0.29)	102.20 (4.31)
Na	138.64 (2.48)	138.57 (1.96)	138.85 (1.51)	138.50 (1.32)	140.33 (1.15)	138.90 (1.67)
CO2	28.22 (1.71)	27.04 (2.17)	28.15 (2.22)	27.73 (3.24)	30.00 (1.73)	27.38 (3.35)
Hgb	13.53 (0.95)	12.85 (1.90)	13.47 (0.94)	13.18 (1.73)	13.57 (0.23)	12.37 (0.85)
RDW	13.35 (0.86)	13.92 (1.28)	13.68 (1.27)	14.61 (1.41)	14.53 (0.29)	13.32 (0.29)
Hct	40.40 (2.80)	39.67 (4.63)	41.58 (2.71)	39.59 (4.78)	42.75 (2.42)	37.19 (2.63)
Chol-HDL	40.57 (14.00)	31.36 (12.69)	29.48 (14.32)	26.05 (10.61)	41.33 (1.15)	31.10 (9.34)
Chol-LDL	97.37 (25.15)	75.92 (28.82)	69.92 (22.46)	90.41 (43.48)	85.33 (21.36)	87.80 (30.47)
Chol-Non-HDL	125.94 (32.09)	104.37 (29.73)	85.69 (13.06)	114.86 (38.62)	113.67 (6.35)	111.20 (29.41)
Chol	182.32 (39.06)	146.16 (34.23)	127.19 (29.85)	143.55 (36.49)	153.83 (13.28)	147.60 (22.19)

### Analysis of retinal and peripapillary microvasculature revealed correlation of microvascular abnormality with severity of retinopathy

Patients with diabetic retinopathy experience various levels of vasculopathy, including nonperfusion and neovascularization, which can be visualized on OCTA imaging. We quantified the effect of the disease on the retinal layer by the correlation between structural thickness, derived from OCT imaging, and vascular changes, derived OCTA imaging.

**Figure 4:**
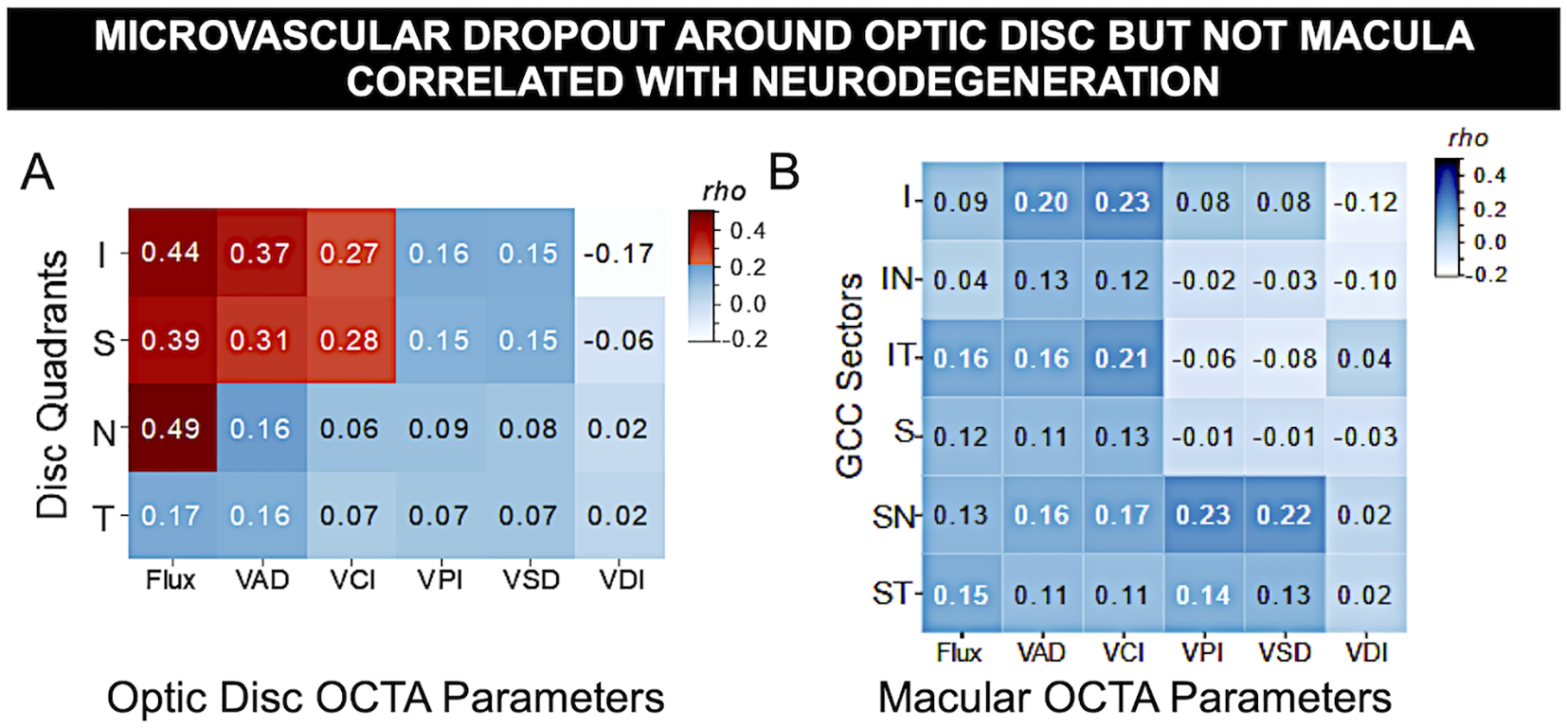
Microvascular dropout around optic disc but not macula correlated with neurodegeneration. Spearman’s *rho* correlation matrix of OCT vs. OCTA measurements in DM eyes revealed significant correlation of OCTA vascular flux, vessel area density, and vessel complexity index with OCT thickness of optic nerve unmyelinated axons due to neurodegeneration but not with inner retinal macular ganglion cell complex, where macular edema often occurs. **(A)** Optic disc peripapillary retinal fiber layer (pRNFL) quadrants thickness vs. 6 peripapillary microvascular parameters (I, inferior; S, superior; N, nasal; T, temporal). **(B)** Macular ganglion cell complex sector thickness vs. 6 macular OCTA measurements (IN, inferonasal; IT, inferotemporal; SN, superonasal; ST, superotemporal sectors). Red: statistical significance after Bonferroni correction for multiple comparisons, blue: non-significance. The gradient of color illustrates correlation strength.

The results revealed that in patients of more severe DR, optic disc measurements from OCT and OCTA showed comparatively stronger correlations as compared to both patients with less severe DR, and to macular measurements. Specifically, the results indicated worsening flux, vessel area density and reduced vessel complexity that is moderately correlated with the inferior and superior quadrants in optic disc, and with worsening diabetic retinopathy severity, likely due to known microvascular pathology such as nonperfusion, microaneurysms, and loss of pericytes. Spearman’s rho analysis revealed significant correlations between certain optic disc and RNFL measurements: namely flux, vessel area density and vessel complexity index within the inferior and superior quadrants. Spearman’s rho correlation coefficients for flux, vessel area density and vessel complexity index were 0.44, 0.37 and 0.27, and 0.39, 0.31 and 0.28 for inferior and superior quadrants respectively (**Figure 4**, **Table 2**). Additionally, RNFL thickness within the nasal quadrant was also significantly correlated with flux (r = 0.49). These significant correlations indicate relative thinning of the OCT and OCTA flux, VAD, and VCI measurements in the superior and inferior quadrants – patterns consistent with a combination of structural and vascular low in retinal degeneration in DM.

**Figure 5:**
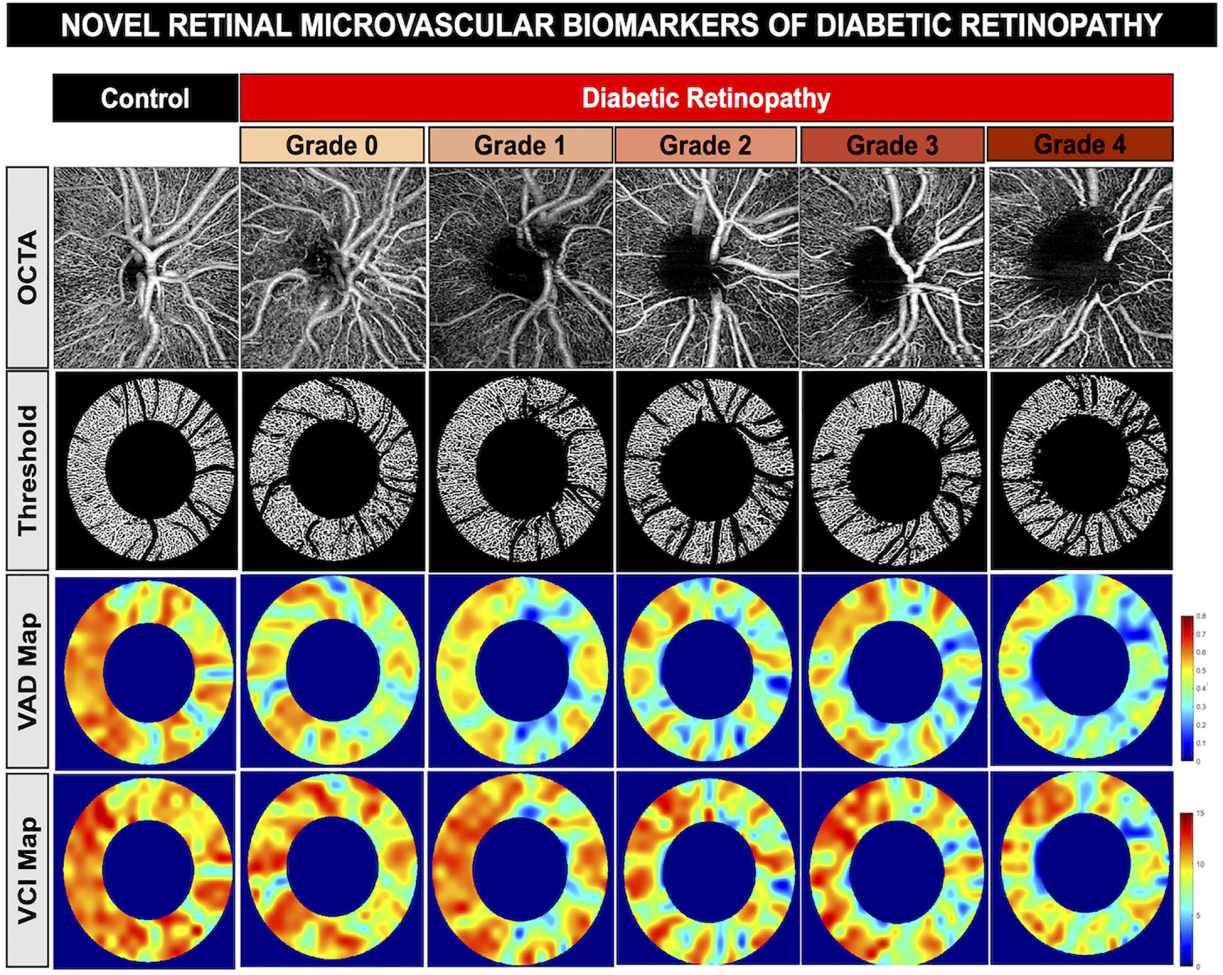
Novel retinal Microvascular biomarkers of diabetic retinopathy. Representative optical coherence tomography angiography of the optic disc showing worsening of vessel density and reduced vessel complexity with worsening of diabetic retinopathy. First row: OCTA images of a healthy control patient and diabetic retinopathy patients in increasing severity from grade 0 to grade 4. Second row: vessel skeleton and annulus of OCTA images generated from custom MatLab script. Third row: Vessel area density (VAD) heat map, indicating areas of high vessel area density (red) to areas of low vessel area density (blue). Fourth row: Vessel complexity index (VCI) heat map, including areas of high vessel complexity (red) to areas of low vessel complexity (blue).

Worsening of vascular changes in more severe DR are seen including microvascular dropout of the optic disc, but not the macula (**Figure 5**). Despite moderate correlation between sectoral OCTA measurements with RNFL, the results did not reveal any significant relationships between macular GCC and OCTA (Spearman’s rho coefficients ranged from 0.02 to 0.23, with all p-values above Bonferroni-corrected significance level). This indicates that microvasculopathy is not reflected in basic thickness measurements. Overall, the results between OCTA and optic disc thickness illustrates an association between OCT and OCTA measurements.

The fact that this result is observed in more severe DR patients and only in optic disc indicates that patients and retinal structures are not impacted uniformly. One possible explanation for why that macular structures do not share the same relationship between OCT and OCTA measurements could be due to the fact that they are not affected yet, due to disease severity (disease has not progressed enough) or time (within the same disease severity, macular regions are only observable after a period of time).

## Discussion

Analysis of 2,201 eye clinic encounters revealed that 91% of eyes in patients with diabetes followed by ophthalmologists had good visual prognosis, with maintenance of visual acuity better than 20/40 over 4-7 years. Analysis of 44,833 blood laboratory data points revealed that patients with more severe DR exhibit evidence of nephropathy, so renal function should be closely monitored. Even borderline abnormal renal function may be early indicator of irreversible multisystem end organ damage. OCTA analysis provided detailed understanding of papillary and retinal microvascular damage in different severities of DR. We found that there is evidence of microvascular drop out even in those with DR grade 0 and that not surprisingly these vascular changes worsen in more severe DR, supporting greater use of this technology in care of patients with DR. While OCTA is likely the most sensitive and noninvasive method to identify microvascular abnormality, this technique and proper analysis are not yet readily available at point-of-care locations to impact patient care. However, we found that optic disc OCTA was most correlated with peripapillary retinal nerve fiber layer thickness in the superior and inferior quadrants – measurements that are commonly available.

There is a well-known clinical association between DM and chronic kidney disease, as the microvascular changes in the superficial capillary plexus also reflects decreased renal perfusion and renal function (14–16). Our analyses revealed that elevated hyperglycemia and evidence of renal failure (elevated blood urea nitrogen, increased creatinine, and decreased eGFR) are more common in those with moderate to severe DR. Patients with severe DR (DR4) were found to have significantly decreased renal function compared to healthy controls and mild and moderate DR patients. Diabetic retinopathy and nephropathy are common complications of DM (14–17), which are consistent with our findings.

Optic disc and macular thickness on OCT and vascular parameters on OCTA correlated with severity of diabetic retinopathy and can be found even in those without clinical evidence of diabetic retinopathy based on examination and fundus color imaging (DR0). This data revealed that worsening severity of diabetic retinopathy is associated with decreased vessel area density and decreased vessel complexity index. This could indicate that patients with progressive DR may be experiencing microvascular nonperfusion, hemorrhages, or overall vascular death (1, 4).

The severity of diabetic retinopathy is graded by evidence of vasculopathy (18, 19). Vascular analysis using OCTA of different layers of the retina revealed significant correlation of neurodegeneration of the unmyelinated optic nerve axon with 3 vascular parameters (vessel flux, vessel area density, and vessel complexity index) in the superior and inferior peripapillary quadrants – the areas most significantly affected in DM. Although OCT and OCTA present as powerful tools to detect microvascular changes and vasculopathy in the superficial capillary plexus (20, 21), our data revealed that there were no significant changes or vasculopathy correlation in the macula. The literature on OCT thickness in DM is highly varied (22–24), so quantification of microvascular changes may be more sensitive for detecting DM-related pathology than that of overall thickness, since it reflects changes due to neovascularization and macular edema (23–25). In a related study conducted by Fernandez-Espinosa et al. showed similar results in that patients with diabetes without evidence of diabetic macular edema had decreased vascular density in the superficial capillary plexus of the retina (26).

Neuropathy, a known complication of diabetes, has been found in recent literature to be a cause of retinal degeneration in patients with diabetes, which precedes the early microvascular changes noted in patients in this study, as evidenced by OCTA. This new data supported by findings reported by Cipres et al. further support the need for early vascular imaging via OCTA in patients with diabetes to detect microvascular changes in the retina to prevent the progression of retinal disease (27).

Our study’s novelty in showing both microvascular changes with OCTA in combination with longitudinal blood chemical data to illustrate disease progression with these parameters indicates an more individualized approach for improved clinical care in patients with diabetes. Moreover, a related study conducted by Sun et al. showed that OCTA serves a promising role in quantifying early microvascular changes in the retina in patients with diabetes (28). This study correlates well with findings in this study, as the OCTA quantification allowed us to identify patterns of microvascular changes in the retina in the early stages of diabetic retinopathy, indicating that this novel imaging technique shows promising clinical advantage to improve care for patients with diabetes.

Limitations to this study include inconsistent ophthalmic imaging with number of follow-up visits and available blood test results ordered based on clinical judgement rather than comprehensive analysis, limiting out ability to detect clinically significant end organ abnormalities.

Overall, this project illuminated the quality ophthalmic care of DM patients at Stanford Byers Institute. The information collected in this study will be used as an informative clinical tool to help improve preventive ophthalmic care for DM patients with routine clinical testing and non-invasive and quick imaging modalities as OCT/OCTA.

## Data Availability

The raw data supporting the conclusions of this article will be made available by the authors, without undue reservation.

## References

[1] Cheung N Mitchell P Wong TY. Diabetic retinopathy. Lancet. (2010) 376:124–36. doi: 10.1016/S0140-6736(09)62124-3 20580421

[2] Cunha-Vaz J Ribeiro L Lobo C. Phenotypes and biomarkers of diabetic retinopathy. Prog. Retin Eye Res. (2014) 41:90–111. 24680929 10.1016/j.preteyeres.2014.03.003

[3] Zarranz-Ventura J Barraso M Alé-Chilet A Hernandez T Oliva C Gascon J. Evaluation of microvascular changes in the perifoveal vascular network using optical coherence tomography angiography (OCTA) in type I diabetes mellitus: a large scale prospective trial. BMC Med Imaging. (2019) 19:91. doi: 10.1186/s12880-019-0391-8 31752726 PMC6873669

[4] Sachdeva MM. Retinal neurodegeneration in diabetes: an emerging concept in diabetic retinopathy. Curr Diabetes Rep. (2021) 21:65. doi: 10.1007/s11892-021-01428-x PMC8668853 34902066

[5] Wang W Lo ACY. Diabetic retinopathy: pathophysiology and treatments. Int J Mol Sci. (2018) 19:1816. doi: 10.3390/ijms19061816 29925789 PMC6032159

[6] Shukla UV Tripathy K. Diabetic retinopathy. In: StatPearls. StatPearls Publishing, Treasure Island (FL (2022). 32809640

[7] Barth T Helbig H. Diabetische retinopathie [Diabetic retinopathy. Klin Monbl Augenheilkd. (2021) 238:1143–59. doi: 10.1055/a-1545-9927 34380155

[8] Bianco L Arrigo A Aragona E Antropoli A Berni A Saladina A. Neuroinflammation and neurodegeneration in diabetic retinopathy. Front Aging Neurosci. (2022) 14:937999. doi: 10.3389/fnagi.2022.937999 36051309 PMC9424735

[9] van de Kreeke JA Darma S Chan Pin Yin JMPL Tan HS Abramoff MD Twisk JWR. The spatial relation of diabetic retinal neurodegeneration with diabetic retinopathy. PLoS One. (2020) 15:e0231552. doi: 10.1371/journal.pone.0231552 32298369 PMC7161968

[10] Marques IP Ferreira S Santos T Madeira MH Santos AR Mendes L. Association between neurodegeneration and macular perfusion in the progression of diabetic retinopathy: A 3-year longitudinal study. Ophthalmologica. (2022) 245:335–41. doi: 10.1159/000522527 PMC9393829 35158351

[11] Kim K Kim ES Kim DG Yu SY. Progressive retinal neurodegeneration and microvascular change in diabetic retinopathy: longitudinal study using OCT angiography. Acta Diabetol. (2019) 56:1275–82. doi: 10.1007/s00592-019-01395-6 31401734

[12] Lowe HJ Ferris TA Hernandez PM Weber SC. STRIDE–An integrated standards-based translational research informatics platform. AMIA Annu Symp Proc. (2009), 391–5. PMC2815452 20351886

[13] Touzeau O. Calculs de l’acuité visuelle moyenne et de la variation d’acuité visuelle à partir d’une échelle décimale [Calculating the mean visual acuity and the change in visual acuity with a decimal acuity chart. J. Fr Ophtalmol. (2003) 26:586–90. 12910197

[14] Faselis C Katsimardou A Imprialos K Deligkaris P Kallistratos M Dimitriadis K. Microvascular complications of type 2 diabetes mellitus. Curr Vasc Pharmacol. (2020) 18:117–24. doi: 10.2174/1570161117666190502103733 31057114

[15] Saini DC Kochar A Poonia R. Clinical correlation of diabetic retinopathy with nephropathy and neuropathy. Indian J Ophthalmol. (2021) 69:3364–8. doi: 10.4103/ijo.IJO_1237_21 PMC8725070 34708806

[16] Yang J Liu Z. Mechanistic pathogenesis of endothelial dysfunction in diabetic nephropathy and retinopathy. Front Endocrinol (Lausanne). (2022) 13:816400. doi: 10.3389/fendo.2022.816400 35692405 PMC9174994

[17] King RJ Harrison L Gilbey SG Santhakumar A Wyatt J Jones R. Diabetic hepatosclerosis: another diabetes microvascular complication. Diabetes Med. (2016) 33:e5–7. doi: 10.1111/dme.2016.33.issue-2 26315331

[18] Heng LZ Comyn O Peto T. Diabetic retinopathy: pathogenesis, clinical grading, management and future developments. Diabetes Med. (2013) 30:640–50. doi: 10.1111/dme.2013.30.issue-6 23205608

[19] Cicinelli MV Cavalleri M Brambati M Lattanzio R Bandello F. New imaging systems in diabetic retinopathy. Acta Diabetol. (2019) 56:981–94. doi: 10.1007/s00592-019-01373-y 31203437

[20] Johannesen SK Viken JN Vergmann AS Grauslund J. Optical coherence tomography angiography and microvascular changes in diabetic retinopathy: a systematic review. Acta Ophthalmol. (2019) 97:7–14. doi: 10.1111/aos.2019.97.issue-1 30238633

[21] Shen C Yan S Du M Zhao H Shao L Hu Y. Assessment of capillary dropout in the superficial retinal capillary plexus by optical coherence tomography angiography in the early stage of diabetic retinopathy. BMC Ophthalmol. (2018) 18:113. doi: 10.1186/s12886-018-0778-2 29739379 PMC5941753

[22] Shin YI Nam KY Lee SE Lee MW Lim HB Jo YJ. Lee, S.E. et al. Peripapillary microvasculature in patients with diabetes mellitus: An optical coherence tomography angiography study. Sci Rep. (2019) 9:15814. doi: 10.1038/s41598-019-52354-8 31676848 PMC6825207

[23] Tang FY Ng DS Lam A Luk F Wong R Chan C. Determinants of quantitative optical coherence tomography angiography metrics in patients with diabetes. Sci Rep. (2017) 7:2575. doi: 10.1038/s41598-017-02767-0 28566760 PMC5451475

[24] Vujosevic S Muraca A Gatti V Masoero L Brambilla M Cannillo B. Peripapillary microvascular and neural changes in diabetes mellitus: an OCT-angiography study. Invest Ophthalmol Vis Sci. (2018) 59:5074–81. doi: 10.1167/iovs.18-24891 30357402

[25] Han Y Wang X Sun G Luo J Cao X Yin P. Quantitative evaluation of retinal microvascular abnormalities in patients with type 2 diabetes mellitus without clinical sign of diabetic retinopathy. Transl Vis Sci Technol. (2022) 11:20. doi: 10.1167/tvst.11.4.20 PMC9034707 35446407

[26] Fernández-Espinosa G Boned-Murillo A Orduna-Hospital E Díaz-Barreda MD Sánchez-Cano A Bielsa-Alonso S. Retinal vascularization abnormalities studied by optical coherence tomography angiography (OCTA) in type 2 diabetic patients with moderate diabetic retinopathy. Diagnostics (Basel). (2022) 12:379. doi: 10.3390/diagnostics12020379 35204470 PMC8871460

[27] Ciprés M Satue M Melchor I Gil-Arribas L Vilades E Garcia-Martin E. Retinal neurodegeneration in patients with type 2 diabetes mellitus without diabetic retinopathy. Arch Soc Esp Oftalmol (Engl Ed). (2022) 97:205–18. doi: 10.1016/j.oftale.2022.02.009 35523467

[28] Sun Z Yang D Tang Z Ng DS Cheung CY. Optical coherence tomography angiography in diabetic retinopathy: an updated review. Eye (Lond). (2021) 35:149–61. doi: 10.1038/s41433-020-01233-y PMC7852654 33099579

